# Supplementary material for: Exploring the Heterogeneity of Cancer-Associated Fibroblasts via Development of Patient-Derived Cell Culture of Breast Cancer
Source: Int J Mol Sci. 2025 Aug 12;26(16):7789. doi: 10.3390/ijms26167789 (PMC12386620; doi:10.3390/ijms26167789)
Supplement: Supplementary file 1 [file ijms-26-07789-s001.zip › ijms-3730476-supplementary.pdf]

Supplementary Materials:.

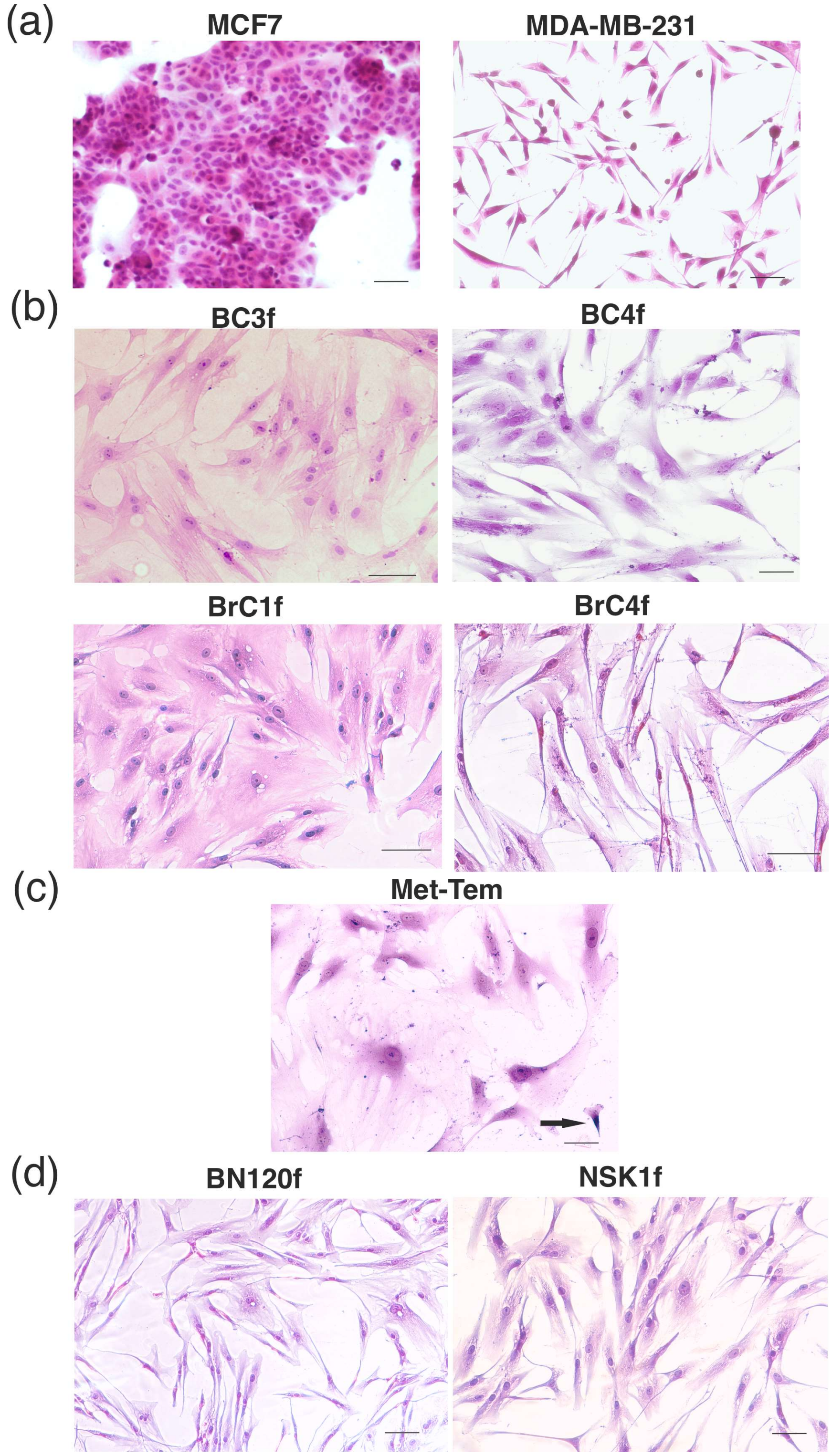

Figure S1. Cell morphology of patient-derived cultures of breast cancer and normal tissue. **(a)** Epithelial-like MCF-7 and mesenchymal-like MDA-MB-231 breast cancer cell line; **(b)** patient-derived fibroblast culture from breast tissue; **(c)** metastatic patient-derived fibroblast from brain tissue; **(d)** patient-derived

fibroblast cell culture BN120f from normal breast tissue and NSK1 from the eyelid tissue. Hematoxylin and eosin staining. Magnification 20x. Black arrow—epithelium-like cells, light arrow—nuclei.

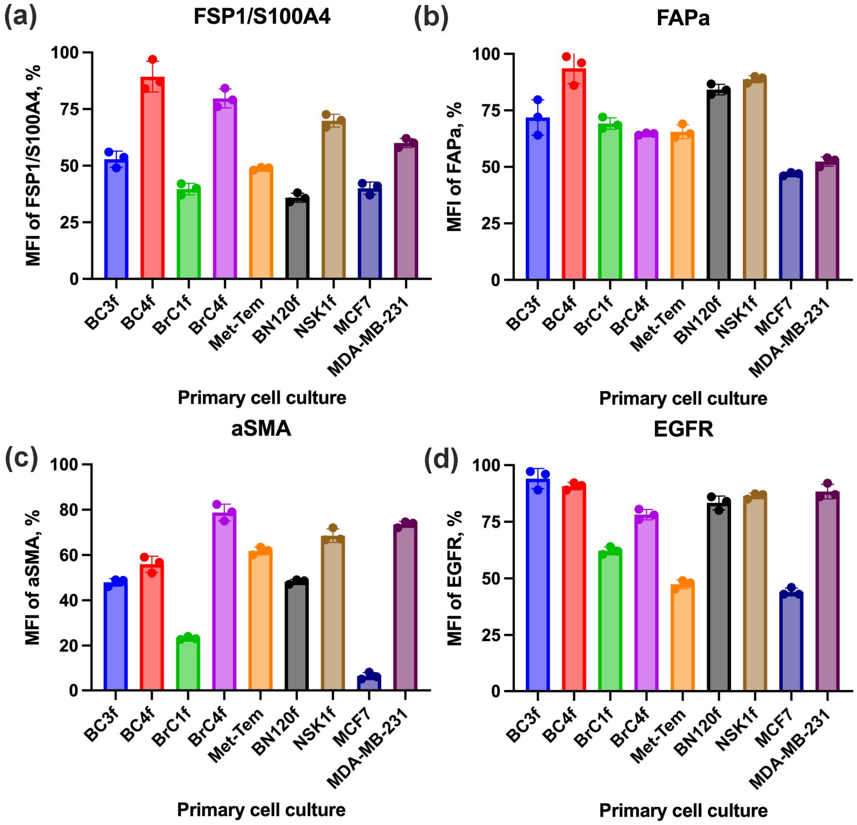

Figure S2. The bar graphs show the mean fluorescence intensity (MFI) obtained for each of the proteins in cells. MFI of **(a)** FSP1/S100A4 and F-actin; **(b)** FAPα; **(c)** αSMA; and **(d)** EGFR in fibroblast. Data are mean ± standard deviation of three different fields of view. Normal cells (BN120f and NSK1) and immortalized BC line used as control.

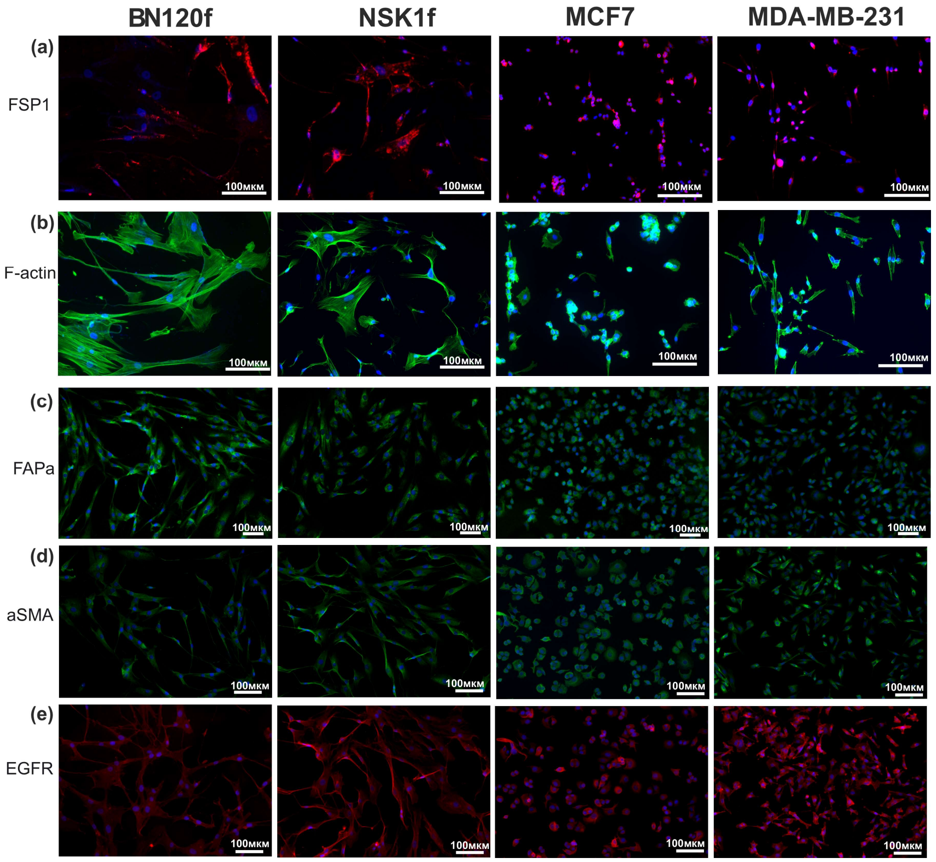

Figure S3. Immunofluorescence staining of markers of cancer-assisted fibroblast in normal cells and immortal cell line. Expression of **(a)** FSP1/S100A4 (red signal) and F-actin (green signal); **(b)** FAPα (green signal); **(c)** αSMA (green signal) and EGFR (red signal) in fibroblast. Hoechst 33342 staining nuclei (blue). Magnification 10x, 20x.

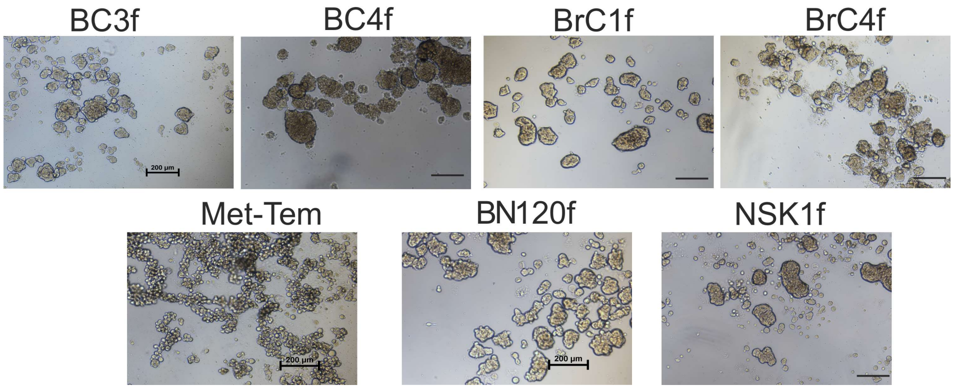

Figure S4. Potential fibroblast to cell aggregation in 3D formats.

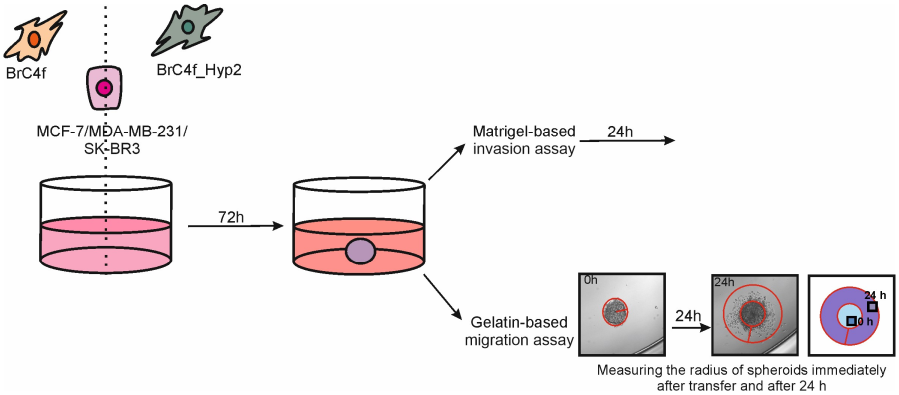

Figure S5. Schematic representation illustrating the study cells' potential for invasion and migration. The cells are located on a model of heterotypic 3D-2 spheroids.
